# Preferred Interaction Styles for Human-Robot Collaboration Vary Over Tasks With Different Action Types

**DOI:** 10.3389/fnbot.2018.00036

**Published:** 2018-07-04

**Authors:** Ruth Schulz, Philipp Kratzer, Marc Toussaint

**Affiliations:** Machine Learning and Robotics Lab, Institut für Parallele und Verteilte Systeme, Stuttgart University, Stuttgart, Germany

**Keywords:** HRI, cooperation, PR2, joint action, collaboration, shared autonomy, robotics, interaction

## Abstract

How do humans want to interact with collaborative robots? As robots become more common and useful not only in industry but also in the home, they will need to interact with humans to complete many varied tasks. Previous studies have demonstrated that autonomous robots are often more efficient and preferred over those that need to be commanded, or those that give instructions to humans. We believe that the types of actions that make up a task affect the preference of participants for different interaction styles. In this work, our goal is to explore tasks with different action types together with different interaction styles to find the specific situations in which different interaction styles are preferred. We have identified several classifications for table-top tasks and have developed a set of tasks that vary along two of these dimensions together with a set of different interaction styles that the robot can use to choose actions. We report on results from a series of human-robot interaction studies involving a PR2 completing table-top tasks with a human. The results suggest that people prefer robot-led interactions for tasks with a higher cognitive load and human-led interactions for joint actions.

## 1. Introduction

As robots become more common in industrial and private settings, they need to be able to interact collaboratively with humans. Research in the Human-Robot Interaction field often draws on previous research on human interactions, but there are still many unanswered questions regarding how people want to interact with collaborative robots. How should the robot choose which action to perform? When should the robot watch or ask questions rather than acting? When do people appreciate verbal assistance from the robot?

In situations in which humans and robots collaboratively work in close proximity, the actions of the agents need to be coordinated so that the human and the robot can efficiently work together without hindering each other. Previous work has focused on different coordination mechanisms that can be used for a human and a robot to communicate about which actions should be performed next (Lallée et al., 2013; Devin et al., 2017) and designing interactions with different roles with respect to the planning and execution of actions (Shah et al., 2011; Baraglia et al., 2016; Roncone et al., 2017). Key mechanisms for achieving effective coordination include joint attention, action observation, task-sharing, action coordination, and perception (Mutlu et al., 2013). A variety of robot systems have been designed to improve specific elements of human-robot interaction.

Gaze cues have been shown to enable more effective joint attention and to improve both task performance and the perception of the robot for a task in which a robot instructs participants to sort objects based on color and shape (Mutlu et al., 2013) and to reduce task completion times when a robot instructs participants which target block to move (Boucher et al., 2012). Gaze information has also been used to indicate current state and intentions so that a robot can identify the best action to assist a human assembling a LEGO object based on a memorized plan (Sakita et al., 2004). Additionally, task performance and user experience can be improved when the robot monitors task progress for an assembly task and provides appropriate feedback to rectify problems (Mutlu et al., 2013). Social cues, such as nods, gestures, and gaze, have been shown to improve teamwork efficiency and robustness, with people able to coordinate their behavior with a robot when performing a button naming and pressing task (Breazeal et al., 2005). The cognitive load of the human partner can be reduced when the robot assists in the planning part of the task. When the robot assists in planning for collaboratively building a stool, lower task completion times are achieved than for when the human does all of the planning (Roncone et al., 2017). Communicating about shared plans using gaze and speech aids effective collaboration for a task in which a human and robot work together to uncover a toy that is covered by a box (Lallée et al., 2013). Specifically, a robot without a joint plan resulted in lower cooperation and success than when the robot had a joint plan that it communicated to the human with either language, gaze, or both. A way to implicitly communicate about a shared plan is to simply make the correct action at the appropriate time, thereby reducing verbalizations. A robot that makes the right decision at the right time during a block world construction task, with a conflict regarding whether the human or the robot should place a particular block, showed improved efficiency, fluency, and acceptability (Devin et al., 2017). Perspective taking, which is vital for efficient human-robot interactions that use language to describe spatial relationships, has been successfully integrated into a cognitive architecture to assist in accurate understanding of navigation instructions (Trafton et al., 2005).

Another strategy for improving coordination between humans and robots is for both the robot and the human to adapt to each others' preferences and abilities. For the robot, this can be achieved by forming user profiles with respect to a variety of different behaviors (Rossi et al., 2017). These profiles may include how the human physically moves with respect to where, how, and how fast movements should be made; what decisions the human makes cognitively with respect to planning, perspective taking, and collaborating; and how the human acts socially with respect to reciprocal behaviors, personality, and non-verbal cues. In practice, this can be performed by keeping track of interactions with the human over time and modeling human knowledge so that an appropriate level of guidance can be provided, for example by determining the current knowledge of a human for a cooking task (Milliez et al., 2016). Alternatively, a robot can use a learning algorithm to update action selection strategies over iterations of interactions. In a study investigating consecutive assembly of a toolbox, the feedback from the human partner decreased over time, showing that the robot was able to learn user preferences (Munzer et al., 2017b). In a study involving a set of interaction behaviors, a robot was able to use gaze and movement as signals of human comfort to adjust interaction distances, gaze, and motion speed and timing to individual preferences with moderate success (Mitsunaga et al., 2008). The robot can also act in a way that allows the human to efficiently adapt to the robot, for example, by showing the human that it cannot see an object by knocking it over or cannot hold a heavy object by dropping it (Nikolaidis et al., 2017). While such actions are obviously not optimal for completing the current task, they may improve the successful completion of future tasks.

Interaction style, or how a robot interacts with the human with respect to autonomous action or command-driven action, can also affect the efficiency of interactions and perceptions about the robot. There are three main styles for these interactions: autonomous, human-led, or robot led interactions. Baraglia et al. (2016) found that a robot that proactively helps the human or one that is controlled by the human is preferred to one that waits before proposing help for a table-top placement task, with better team fluency and higher subjective ratings. In simple situations, proactive action selection can speed up task completion, and in complex situations, it can aid in the robot's understanding of the human's intentions by provoking a reaction by the human to confirm or disprove the information (Schrempf et al., 2005). A robot using Chaski, a robot plan execution system, was able to perform better than a robot that was verbally commanded by the human team mate for a collaborative task involving collecting blocks and assembling structures (Shah et al., 2011). Munzer et al. (2017a) compared a semi-autonomous robot with an instructed robot, and found that the semi-autonomous robot was preferred, and that for the toolbox assembly task investigated, people would prefer a robot with even more autonomy. A robot system has been designed for taking a leader or a follower role for solving a physical labyrinth game with preliminary results indicating that the role taken by the robot may affect human perceptions of the safety and intelligence of the robot (Beton et al., 2017). A robot system has also been designed for flexible execution of collaborative tasks by either leading the interactions or acting as an assistant for a task involving cleaning furniture (Fiore et al., 2016). This system is an example of a robot able to switch between leading or following which may be desired behavior for a collaborative robot.

During collaborative task executions the agents need to work together on a task specified by a shared plan. The design of the task is a key consideration for human-robot interaction. Task design ranges from simple tasks, such as removing objects from a table (Nikolaidis et al., 2017), to complex tasks, like assembling structures (Munzer et al., 2017b). While simple tasks do not have a fixed order of action selection a complex task can have dependencies of actions and can even require the agents to do something at the same time, for example, one agent might have to lift an object while the other agent does something below (Lallée et al., 2013). There are many features of the task that may affect the interaction between humans and robots, including the number of agents, the environment of the task, the mobility of the agents, the position of the agents, the development of a shared plan, the knowledge of the task, and the level of communication. We consider two dimensions to be of particular interest for task design: *fixed vs. any order actions* and *independent vs. joint action*.

In previous work, tasks involving *any order actions* are typically clearing (Nikolaidis et al., 2017) or cleaning (Fiore et al., 2016) tasks, where each action can be completed independently. There may be some actions that can only be performed by one of the agents, for example due to lack of mobility of the robot. Tasks in which at least some actions must be completed in a *set order* include construction of furniture (Roncone et al., 2017), building structures (Devin et al., 2017), cooking (Milliez et al., 2016), and table-top placement tasks (Baraglia et al., 2016). Many actions in previous studies are *independent actions*, meaning that a single agent can perform the action, for example moving an object (Baraglia et al., 2016; Nikolaidis et al., 2017) or collecting pieces for later construction (Shah et al., 2011). The alternative is *joint action*, where two agents are needed to successfully perform the action. In previous work, joint action typically involves the robot holding a part while the human performs the construction, for example of the toolbox (Munzer et al., 2017b) or furniture (Roncone et al., 2017). Another example of joint action is where one agent picks up a box so that the other agent can retrieve the toy hidden underneath (Lallée et al., 2013).

While previous work has typically found that autonomous action is more efficient, it is unclear whether humans always prefer autonomous action, or whether there are some situations for which explicit commands by either the human or the robot are preferred. We believe that the type of task being executed will make a difference with respect to how efficient the collaborations are with different styles of interaction. The interaction style and task completed may also affect how the collaboration is perceived by the human with respect to efficiency, comfort, safety, and fluency.

In this work, we study different interaction styles for a robot to use when interacting with a human to complete table-top tasks. We aim to identify situations in which different interaction styles are preferred. We have conducted a series of three experiments investigating human-robot interaction for collaborative table-top tasks. In the first of these experiments we explore five different interaction strategies on a table-top blocks construction task. In the second experiment we chose three interaction strategies (Autonomous, Human-Commands, and Robot-Commands) to test on four different tasks which differ in whether actions may be completed in any order or a fixed order, and whether independent or joint action is required (Sort, Stack, Build, Balance). In the final experiment, we designed an Information interaction strategy based on our findings about human preferences in particular situations and compare it to the Autonomous strategy over the same four tasks.

In the following section we first discuss task design and interaction styles for human-robot interaction. We then present our robot system for performing collaborative table-top tasks, the experimental design for the series of experiments on interaction styles over a variety of tasks, the results, and a discussion of the experiments. The results confirm previous studies finding that Autonomous action is more efficient for all tasks. However, we have also identified situations in which human partners prefer more control or less cognitive demand, and have shown that, respectively, human-led and robot-led interactions are preferred in these situations.

This paper extends our previous work on interaction styles (Schulz et al., 2017).

## 2. Interactions

One domain in which collaborative robots can be used is on-table tasks. In a collaborative on-table task, agents manipulate objects on a table to complete a task. The robot needs to be able to perceive the table and objects and be able to manipulate the objects. Ideally, the human and the robot can work out a strategy to complete the task efficiently without hindering each other. In this section, we discuss both our task design and the interaction styles used by the robot to determine how and when to act.

### 2.1. Task design

In developing robot systems for studying human-robot interactions, a key consideration is the design of the task to be collaboratively completed by the human and the robot. The ideal task is sufficiently complex to allow interesting collaborative actions to be investigated, while remaining simple enough so that a useful analysis of the interactions can be performed. There are many features of the task that may affect the interaction between humans and robots, including the number of agents, the environment of the task, the mobility of the agents, the position of the agents, the development of a shared plan, the knowledge of the task, and the level of communication.

In our setup the human sits on a chair on one side of the table. The robot is located on the opposite side of the table. Having the agents on opposite sides of the table allows freedom in moving and positioning arms without hindering the other agent. Moreover, the counterpart is clearly visible so that actions can be seen early and a large area of the table is covered. Objects of different shapes, sizes, and colors can be placed on the table. Both agents can manipulate objects on the table, for example, to place them at specific positions. Both agents have knowledge about the target state and can determine what actions need to be completed to reach this state. We have designed a set of tasks that vary across two dimensions: *order of actions* and *independent vs. joint action*.

*Order of Actions:* Tasks can have different dependencies between subtasks. For example, the subtasks can have relations that enforce a fixed order of actions. These dependencies can mean that one action must be completed before another action, influencing the order of action selection. Consider a task to place blocks in a predefined target structure. Three tasks that vary in the constraints of actions are (1) building a tower of three blocks, (2) placing three blocks in a row across the table, and (3) building a bridge with two blocks on the table and a larger block on top of those two blocks. For the tower, a fixed order of actions is required to accomplish the task. For the row of blocks, there is no relation between the actions and they can be scheduled in any order. For the bridge, a partially fixed order of actions is required, as the two smaller blocks can be placed in any order, but both must be placed before the larger block.

*Independent vs. joint action:* A further property of on-table tasks is the level of collaboration. For instance, a task involving joint action includes subtasks in which agents have to do specific actions at the same time, such as one holding a tool while another agent attaches objects to the tool. In contrast, a task involving only independent actions can be done by one agent on its own, for example, removing objects from a table. In some situations, agents can execute independent actions concurrently, for example, by removing different objects, thereby reducing the task completion time.

**Figure 1:**
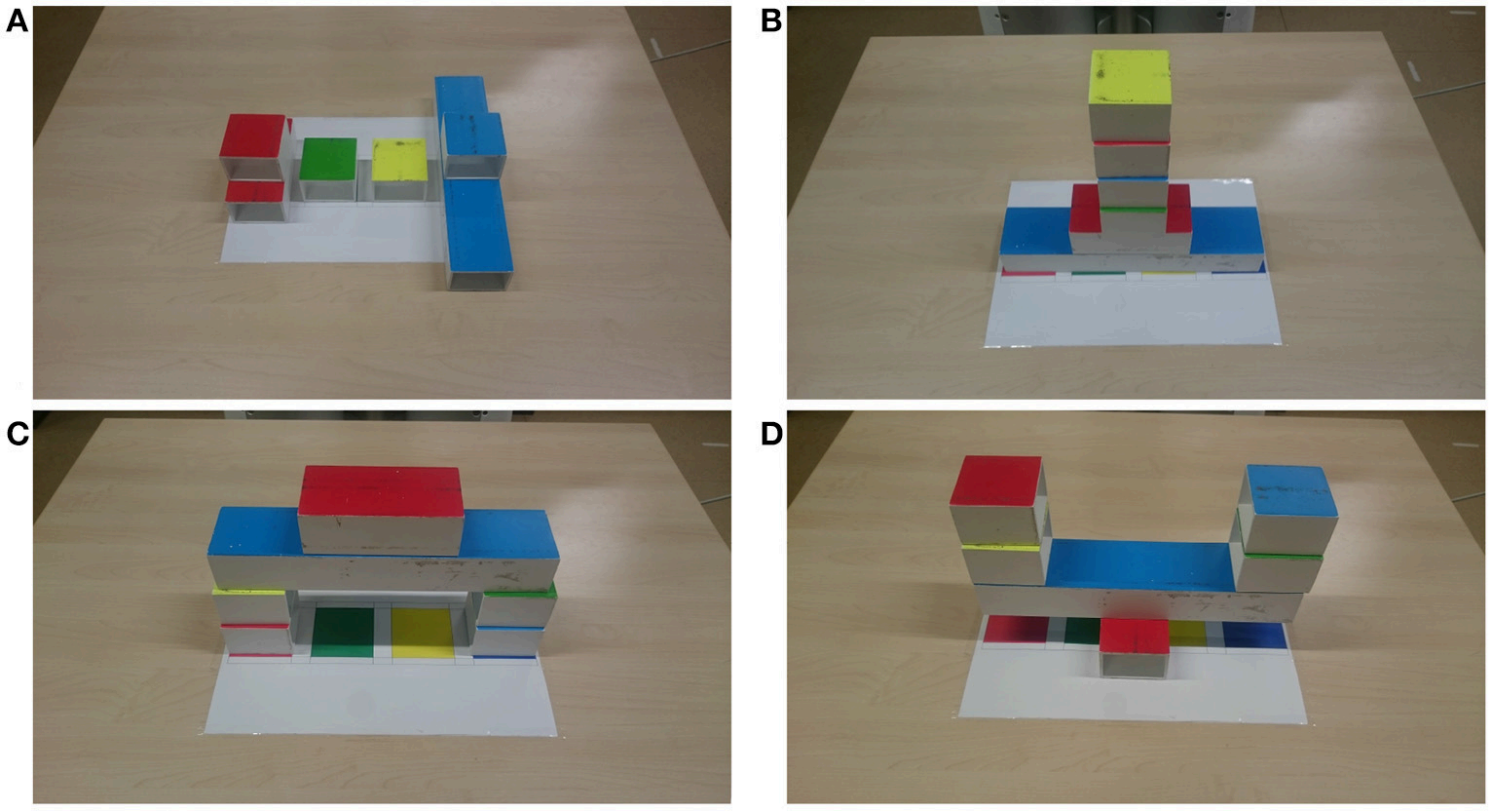
Tasks: **(A)** Sort (any order, independent actions), **(B)** Stack (fixed order, independent actions), **(C)** Build (partially fixed order, independent actions), and **(D)** Balance (fixed order, partially joint actions).

We designed and implemented four tasks in which the human and the robot should place the blocks in the middle of the table in a collaborative manner (see target states in Figure 1). The objective is to place the six blocks in a predefined way to reach a target state known to both the human and the robot. The four tasks are intentionally designed to be very similar, using the same objects and environment, so that they are comparable and controllable. The tasks are named Sort, Stack, Build, and Balance, and differ in whether the comprising actions are *fixed vs. any order* and whether they require *independent vs. joint action*.

- **Sort:** In the Sort task, the blocks should be sorted into four piles, dependent on the color of the block (red, green, yellow, and blue). The blocks can be placed in any order. All actions are independent.
- **Stack:** In the Stack task, the blocks should be placed onto a single stack, with a particular order for which block is at each position in the stack. There is a fixed order for block placement. All actions are independent.
- **Build:** In the Build task, the blocks should be used to build a bridge, with the blocks in particular positions in the bridge. There is a partially fixed order for block placement. All actions are independent.
- **Balance:** In the Balance task, the blocks should be used to build an upside down bridge balancing on the medium block, with the blocks in particular positions in the bridge. The small blocks at the same height in the structure should be placed at the same time so that the structure does not fall down. There is a fixed order for block placement. The first two actions are independent and the balanced blocks require joint action.

The tasks increase in difficulty from Sort, where the placement locations are given by the targets on the table and any order is permitted, to Stack, where the locations are all in the centre of the table and the order is set, to Build, where the locations are partially given by the targets and otherwise set in the information sheet and the order is also partially set, to Balance, where the locations are partially given and otherwise set in the information sheet and the actions must also be co-ordinated.

### 2.2. Interaction styles

The three interaction styles of interest for the current domain are *autonomous action, human-led action*, and *robot-led action*. In this section we describe the seven interaction strategies explored in the studies that are variations on autonomous, human-led, and robot-led interaction styles: Autonomous, Proactive, Reactive, Human-Requested, Human-Commands, Robot-Commands, and Information. Details about the implementation of these strategies are included in section 3.4.

- **Autonomous:** In the Autonomous strategy, both the participant and the robot decide how and when to act in order to complete the task together. The robot obtains the actions that are needed to reach the next sub-task state. If one of the actions can be performed by the robot, the robot autonomously performs the action. If no action is possible, the robot will wait until the state changes.
- **Proactive:** The robot in Proactive mode is similar to the robot in Autonomous mode. However, if none of the actions needed to reach the next subtask state can be performed by the robot, it will start doing an action that needs to be performed later. For example, the robot could grasp an object that needs to be placed on top of a second object that is not yet placed.
- **Reactive:** In Reactive mode, the robot observes the state and monitors the human's actions. If the next subtask state is not reached in a predefined time window, the robot will assume difficulties in performing the action. As a consequence, the robot will perform an action leading to the next subtask, if possible. This mode is similar to the *reactive* mode by Baraglia et al. (2016).
- **Human-Requested:** In the Human-Requested mode, the participant decides how and when to act, and requests help from the robot when needed. The robot observes the state, and when the human requests help, the robot checks which actions reach the next subtask and performs one of them. This mode is similar to the *human-requested* mode by Baraglia et al. (2016).
- **Human-Commands:** In the Human-Commands strategy, the participant decides how and when to act, and also tells the robot how and when to act. The participant specifies which block the robot should move by color and size. For example, “Robot, can you place the small blue block.” The robot obtains the actions that are needed to reach the next sub-task state. If the action is possible, the robot will perform the action. If the block specified is not reachable or not in the set of actions needed to reach the next sub-task state, then the robot waits for another command.
- **Robot-Commands:** In the Robot-Commands strategy, the robot decides how and when to act, and also tells the participant how and when to act. The robot obtains the actions that are needed to reach the next sub-task state. If one of the actions can be performed by the robot, the robot autonomously performs the action. Otherwise, if one of the actions can be performed by the human, the robot will ask the human to perform the action. For example, the robot might say “Can you please place the small red block?” The block is specified by color and size. The participant then works out where to place the block based on the task description.
- **Information:** The robot using the Information strategy chooses actions in the same way as the robot using the Autonomous strategy, however, the robot also tells the participant what it expects the participant to be doing based on its own plan for the task. At each step the robot first checks if it can do anything, and then checks if the participant can do anything either at the same time as the robot or after the robot has placed the current block. The robot then performs an available action and makes an available request of the participant, including asking the participant to indicate when a block should be placed for joint action.

## 3. Materials and methods

In this section, we present the robotic platform, the environment, the perception of the robot, the states and actions of the tasks, and the general experimental protocol.

### 3.1. Robotic platform

**Figure 2:**
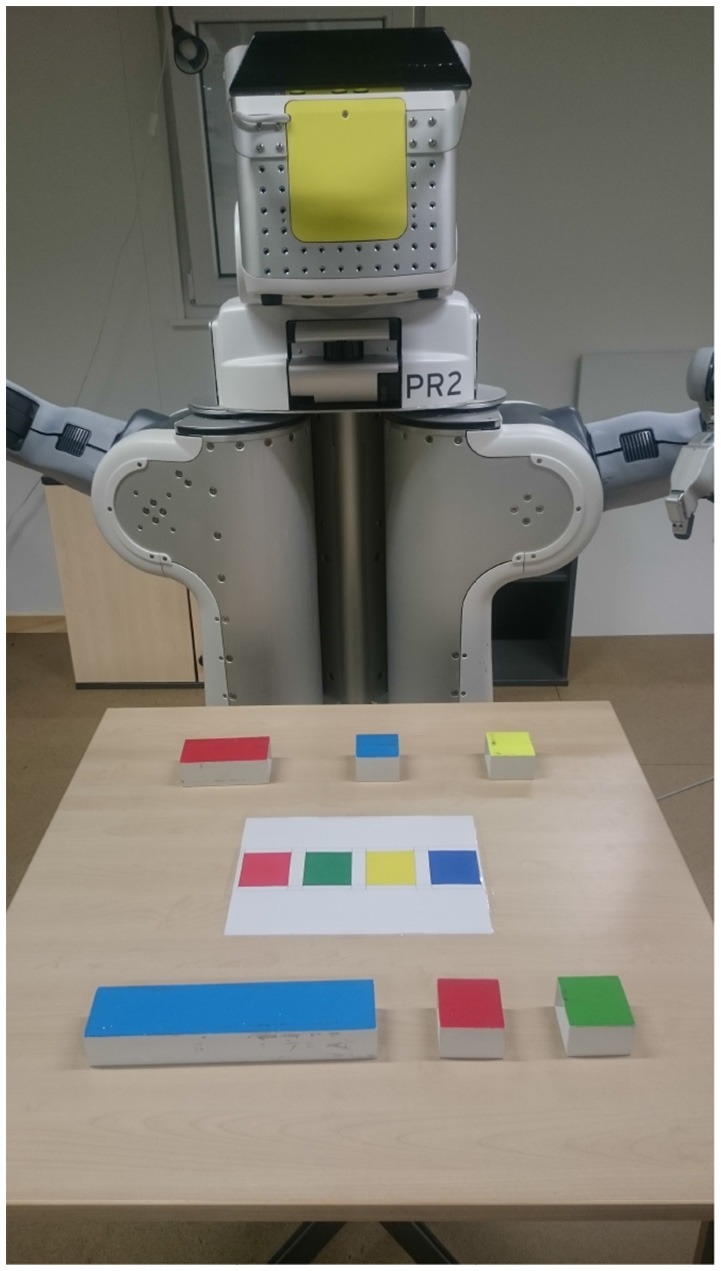
PR2 across the table from the participant, with the blocks and a sheet of paper indicating the target positions for the tasks.

A basic system for collaborative task-execution needs to be able to fulfill specific hardware and software requirements. The system needs to be able to perceive objects on the table and identify the pose of the objects. The system should detect the specific objects and obtain semantic relations, such as which object is placed on top of an other object. As the pose of objects might change due to manipulations of the human, the system should monitor changes and keep track of objects that might be currently undetectable. Furthermore, the system also needs to be able to pick an object and place it in another pose on top of the table. We use the PR2 robotics and research platform (see Figure 2). To perceive objects on the table we use the robot stereo camera, a Microsoft Kinect, that is attached to the head. The head can be panned by 150° and tilted by 115°. As a consequence, the stereo camera can, for example, be aligned to look at the center of the table so that all objects on a table are in the field of view of the sensor. Moreover, the robot has two 7-degree of freedom arms with attached grippers that can be used for on-table pick and place actions. The upper arm has a length of 40 cm and the forearm a length of 32 cm. As a consequence, the robot can reach objects that are approximately up to 70 cm far away from the robot. Furthermore, the robot is equipped with a telescoping spine. This allows to extend the robot torso by 31.5 cm. This helps the robot adapt to different table heights.

### 3.2. Environment

The participant and the robot sit on opposite sides of a table. A camera behind the participant records the study. A smaller table with information sheets and the questionnaire is placed near to the participant. The table between the robot and the participant has a size of 80 cm × 80 cm and a height of 70 cm. Four small blocks of different colors (red, blue, green, and yellow) of the size 6 cm × 6 cm × 4cm, a medium red block of the size 6 cm × 18 cm × 4cm, and a large blue block of the size 6 cm × 27 cm × 4 cm were placed on the table. Three blocks are initially placed close to the robot and three close to the participant. Due to the range of the robot not all of the objects can be picked by the robot. In such situations the human has to pick the object. Accordingly we assume that not all objects can be easily reached by the human. As a consequence, we have three areas of the table: a human-only, a robot-only, and a common area.

### 3.3. Perception

The robot obtains point clouds of tabletop objects by using the depth values of the stereo camera. We use a discretization step to obtain discretized objects with a predefined size and color. This allows a better assignment of detected objects to real world objects. If an object is placed on top of another object, only one point cloud is detected. As a consequence, the system need to keep track of the objects in the scene and adds *below*-relations when another object is placed on top. The perception step is used to update the state of the system, which is used by the robot to select an action.

### 3.4. State and actions

The state, *S*, of the system is described by the state of the table, *S*_*table*_, and the agent states, *S*_*human*_ and *S*_*robot*_. *S*_*table*_ consists of the position and orientation of the objects that are located on the table. Objects located in the hand/gripper of the agent are described by the agent states *S*_*robot*_ and *S*_*human*_. The state *S*_*table*_ can be influenced by both agents while *S*_*robot*_ and *S*_*human*_ only can be influenced by the particular agent.

Both agents can perform actions to manipulate objects on the table or influence the actions of the other agent. Possible actions are to pick up an object, place an object, wait, and communicate. In our system, communication by both the human and the robot is in the form of commands. Both agents follow an action selection policy, π(*S, C*) = *a*, that selects actions based on the current state and command, *C*, if a command has been given, otherwise using an action selection policy, π(*S*) = *a*, that selects actions based only on the current state.

Commands take the form of requesting help or specifying which object the other agent should place next. It is then assumed that the other agent will be able to work out where to place the object. Commands from the robot occur only in the Robot-Commands and Information strategies and are converted from text-to-speech using the ROS sound_play^1^. Speech-to-text is not yet implemented in our system. Commands from the human participants are translated by the researcher into a keyboard command (numbers 1 through 6) selecting the object specified for Human-Commands, or a single command indicating that help has been requested for Human-Requested or that the balanced object should be placed for Information.

Both the robot and the participant know which task is currently being completed, and can determine what actions are needed to complete each task. The robot has complete knowledge of actions to be performed to complete the task from states normally reached when completing the task. The participant has an information sheet with a description of the target state for each of the tasks.

In choosing which action to take, the robot first determines the current state of task completion. It then determines which actions are needed to reach the next sub-task state, and creates a possible-action list. Blocks that are reachable by the robot may be selected for a pick-and-place action. Blocks that are not reachable by the robot may be selected for a command for the human. Depending on the current interaction strategy, the robot may wait for a command, choose a reachable block on the possible-action list for a pick-and-place action, or choose a non-reachable block on the possible-action list for a command for the human.

If there are reachable blocks on the possible-action list, there are several behaviors possible depending on the current strategy. For the Autonomous, Proactive, Reactive, Robot-Commands, and Information strategies, the robot will choose a reachable block on the possible-actions list for a pick-and-place action, noting that the Reactive strategy will first wait for a pre-defined time of inaction. The Information strategy will also determine if the human can execute an action at the same time, choosing a non-reachable block on the possible-action list for a command for the human. In the Human-Requested and Human-Commands strategies, the robot will wait for a command. After a command in the Human-Requested strategy, the robot will choose a reachable block on the possible-action list for a pick-and-place action. After a command in the Human-Commands strategy, if the block indicated in the command is reachable and on the possible-action list, this block will be picked up and placed by the robot. In either case, if the command cannot be executed, the robot will wait for the state to change or for a new command.

If there are no reachable blocks on the possible-actions list, the behaviors possible also depend on the strategy. For the Autonomous, Reactive, Human-Requested, and Human-Commands strategies, the robot will wait for the state to change. For the Proactive strategy, the robot will determine if a pick action can be executed for a later place action. For the Robot-Commands and Information strategy, the robot will choose a non-reachable block on the possible-actions list for a command for the human.

### 3.5. Experimental protocol

A series of three Human-Robot Interaction experiments were performed with the PR2 collaborative robot system described in the previous sections. Here we describe the general experimental protocol, with specifics for the three studies described in the following sections.

Participants were recruited from Stuttgart University through mailing lists. After welcoming our participants, they fill in a consent form and a demographics questionnaire. The interaction strategies and tasks are explained. As the participants need to interact differently with the robot for each strategy, they are informed in advance how to interact with the robot. For the Autonomous, Proactive, Reactive, and Information modes they are told that “both you and the robot will decide how and when to act in order to complete the task together.” For the Information mode, they are also informed that the robot will provide instructions based on its plan for how to complete the task. For the Human-Commands and Human-Requested modes they are told that “you will decide how and when to act, and will also tell the robot how and when to act,” together with details about how to instruct the robot. For the Robot-Commands mode they are told that “the robot will decide how and when to act, and will also tell you how and when to act,” together with details about how the robot will instruct them. Each participant performs the set of tasks with the robot. The blocks are initially placed in the same position for each task. The researcher replaces the blocks in the initial position after the completion of each task. After the interactions, the participant responds to statements about the interactions on a 7-point Likert scale. The questions were inspired by those used by Shah et al. (2011) and Baraglia et al. (2016). A small number of questions are included due to the relatively large number of interaction styles and tasks. The questions were also updated between the different experiments. To gain qualitative feedback about the system, the participant is asked for general comments and improvements for the system after all of the tasks are completed. They are also asked which interaction strategy they preferred both overall and for each task.

## 4. Experiment

A series of three Human-Robot Interaction experiments were performed with the PR2 collaborative robot system described in the previous sections. The first study explores five different interaction styles. The second study compares three interaction styles over four different tasks, and the final study compares two interaction styles over the same four tasks. For each experiment we report the participants' responses to the statements, the task completion times, the rankings of the interaction styles, and general comments. We performed two-tailed unpaired *t*-tests on the completion times in the different conditions and Wilcoxon Signed-Rank tests on the participants' responses to the statements. Due to the small numbers of participants only a small number of significant differences were found, and the ranking of the interaction strategies and the general comments made are more informative for gaining an understanding of how the robot was perceived when it used different strategies.

### 4.1. Interaction styles experiment

In the Interaction Styles experiment, we test five different interaction styles on the one collaborative table-top task: a bridge building task. This task is similar to the Build task described earlier (see Figure 1C), without the medium red block placed on top. The five interaction styles tested were Proactive, Autonomous, Reactive, Human-Requested, and Human-Commands. The order of the interaction styles was randomized. The participants rated the interactions on the following statements:

- The robot and I worked efficiently together
- The collaboration with the robot felt natural
- I was relaxed during the task execution
- The robot was intelligent
- I was surprised by what the robot was doing

The aim of this experiment was to determine preferred interaction styles for a single table-top block construction task. We hypothesize that the Autonomous and Proactive modes are more efficient than the other modes, as found in Baraglia et al. (2016).

#### 4.1.1. Results

We conducted a lab study with 10 participants (2 female) aged between 21 and 51 (*M* = 29.3, *SD* = 9.29). We compared the responses for efficiency, naturalness, relaxedness, intelligence, and surprise (see Table 1). We performed Wilcoxon Signed-Rank Tests on the differences in ratings between the interaction styles. The Proactive strategy is rated as significantly more efficient than the Requested strategy, the Reactive strategy is rated as significantly less efficient than the Proactive, Autonomous, and Human-Commands strategies (*p* ≤ 0.05). The Human-Requested strategy is rated as significantly less natural than the Autonomous strategy, and the Reactive strategy is rated as significantly less natural than the Proactive, Autonomous, and Human-Commands strategies (*p* ≤ 0.05). No statistically significant differences were found for relaxedness, intelligence, and surprise. We compared the difference in rating for efficiency and naturalness between the interaction styles for each participant, and have summarized the average difference in Tables 2, 3, bold values correspond to statistically significant differences. For the Proactive and the Autonomous mode the values are very similar. In our setting the participants often performed their action before the robot finished its action so that it was not necessary to perform an action proactively.

**Table 1:** Rating of the collaboration with the robot as reported by the participants (From 0-*strongly disagree* to 6-*strongly agree*).

	**Proactive**	**Autonomous**	**Requested**	**Commands**	**Reactive**
Efficient	5.30 (0.37)	5.20 (0.42)	4.30 (0.54)	4.90 (0.41)	3.40 (0.52)
Natural	4.40 (0.37)	4.50 (0.34)	3.90 (0.41)	4.30 (0.37)	3.30 (0.33)
Relaxed	5.20 (0.25)	5.10 (0.31)	5.10 (0.23)	5.10 (0.28)	5.00 (0.47)
Intelligent	4.80 (0.29)	4.50 (0.31)	4.60 (0.31)	4.70 (0.52)	3.80 (0.42)
Surprised	1.40 (0.56)	2.30 (0.75)	1.50 (0.62)	1.40 (0.60)	1.50 (0.40)

*Mean (standard error of the mean (SEM))*.

**Table 2:** Comparison of the perception of efficiency between the different interaction styles.

	**Proactive**	**Autonomous**	**Requested**	**Commands**	**Reactive**
Proactive	n/a	0.10 (0.18)	**1.00 (0.47)**	0.40 (0.27)	**1.90 (0.28)**
Autonomous	−0.10 (0.18)	n/a	0.90 (0.55)	0.30 (0.40)	**1.80 (0.36)**
Human Request	−**1.00 (0.47)**	−0.90 (0.55)	n/a	−0.60 (0.45)	0.90 (0.50)
Human Commands	−0.40 (0.27)	−0.30 (0.40)	0.60 (0.45)	n/a	**1.50 (0.31)**
Reactive	−**1.90 (0.28)**	−**1.80 (0.36)**	−0.90 (0.50)	−**1.50 (0.31)**	n/a

*A positive number indicates that the interaction style of the row is rated as more efficient than the interaction style of the column. Bold values indicate statistically significant differences. Mean (SEM)*.

**Table 3:** Comparison of the perception of naturalness between the different interaction styles.

	**Proactive**	**Autonomous**	**Requested**	**Commands**	**Reactive**
Proactive	n/a	−0.10 (0.18)	0.50 (0.27)	0.10 (0.35)	**1.10 (0.28)**
Autonomous	0.10 (0.18)	n/a	**0.60 (0.16)**	0.20 (0.33)	**1.20 (0.33)**
Human Request	−0.50 (0.27)	−**0.60 (0.16)**	n/a	−0.40 (0.40)	0.60 (0.40)
Human Commands	−0.10 (0.35)	−0.20 (0.33)	0.40 (0.40)	n/a	**1.00 (0.21)**
Reactive	−**1.10 (0.28)**	−**1.20 (0.33)**	−0.60 (0.40)	−**1.00 (0.21)**	n/a

*A positive number indicates that the interaction style of the row is rated as more natural than the interaction style of the column. Bold values indicate statistically significant differences. Mean (SEM)*.

Regarding task completion times, the Proactive mode (*M* = 60.40 s, *SEM* = 9.03) and the Autonomous mode (*M* = 64.20 s, *SEM* = 17.55) were the fastest, followed by the Human-Requested mode (*M* = 82.60 s, *SEM* = 22.41) and the Human-Commands mode (*M* = 84.00 s, *SEM* = 33.40). In Reactive mode the task lasted the longest (*M* = 117.90 s, *SEM* = 12.72). Two-tailed unpaired *t*-tests found that completion times for the Proactive strategy were significantly shorter than for Human-Requested, Human-Commands, and Reactive strategies, and that completion times for the Reactive strategy were significantly longer than for all other strategies.

#### 4.1.2. Discussion

The results of the Interaction Styles study confirm our hypothesis that the Autonomous and Proactive modes are more efficient than the other modes for completing the bridge-building task. The most promising interaction styles were Autonomous and Human-Commands. Proactive was found to be very similar to Autonomous for this task and this is likely to be true for most tasks in the domain of building structures with blocks on a table when actions are interleaved between the robot and the participant. In situations in which the robot must wait for the participant to execute several actions, the Proactive strategy may be more efficient than the Autonomous strategy. Human-Commands and Human-Requested were also similar, but Human-Commands was considered more efficient and natural. The Reactive strategy took the longest for task completion, and was considered less efficient and natural than the other strategies. In considering the results from the Interaction Styles study, we have also identified another possible interaction style that involves robot-led interactions: Robot-Commands.

### 4.2. Different tasks experiment

In the Different Tasks study, we test the three promising interaction styles identified in the previous study on four different tasks to determine if there are particular types of actions that are more favorable for certain interaction styles. The four tasks are Sort, Stack, Build, and Balance. The three interaction styles are Autonomous, Human-Commands, and Robot-Commands. The participant has an information sheet describing the interaction strategies and tasks to refer to as needed during the study. Each participant performs the set of four tasks with the robot three times, once for each interaction strategy. The order of the interaction strategies is counterbalanced between the participants. The order of the four tasks is kept the same within each strategy in the order of increasing difficulty: Sort, Stack, Bridge, Balance. The blocks are initially placed with three blocks close to the robot and three blocks close to the participant to maximize interleaving of actions for the tasks. The researcher replaces the blocks in the initial position after the completion of each task. We refined the statements for the participants to rate the interactions to get a better understanding of the interactions. The participants rate the interactions on the following statements:

- I was comfortable working with the robot
- I was confused working with the robot
- The interactions felt natural
- The interactions were fluent
- The interactions were efficient
- The robot was a good partner

The aim of this study is to determine preferred interaction styles for tasks that vary with respect to action order and the independence of actions. For this study, following pilot studies with the authors interacting with the robot using the different interaction styles, we hypothesize that:

- Autonomous interactions are more efficient for all tasks.
- Tasks involving joint action are perceived as more efficient in human-led interactions.
- Tasks with higher cognitive load are perceived as easier in robot-led interactions.
- Participants feel less comfortable in robot-led interactions.

#### 4.2.1. Results

We conducted a lab study with 12 participants (4 female) aged between 21 and 30 (*M* = 25.4, *SD* = 2.71). The average completion time for each task was quickest for the Autonomous strategy (see Tables 4–6). The participants' responses were fairly similar across all tasks and strategies, with less than 2 points difference on average between the responses for the different interaction styles (see Tables 4–6, bold values correspond to statistically significant differences). The Autonomous strategy was preferred over the other strategies by 6 participants overall, but the other strategies were also preferred by some participants overall and for specific tasks (see Table 7).

**Table 4:** Comparison of the Autonomous strategy to the Human Commands strategy for ratings reported by the participants and time taken for task completion in seconds.

	**Sort**	**Stack**	**Build**	**Balance**	**Overall**
Comfortable	0.00 (0.39)	−0.33 (0.48)	0.00 (0.25)	0.00 (0.79)	−0.08 (0.25)
Confused	0.25 (0.81)	0.67 (0.70)	0.42 (0.76)	−0.25 (0.80)	0.27 (0.38)
Natural	0.17 (0.55)	−0.08 (0.51)	0.25 (0.52)	0.00 (0.65)	0.08 (0.27)
Fluent	0.42 (0.38)	−0.25 (0.55)	0.50 (0.36)	0.42 (0.36)	0.27 (0.21)
Efficient	0.42 (0.42)	−0.08 (0.58)	0.08 (0.40)	−0.17 (0.69)	0.06 (0.26)
Good Partner	0.75 (0.46)	−0.08 (0.60)	0.50 (0.50)	0.33 (0.75)	0.38 (0.29)
Time	−**33.83 (9.96)**	−7.92 (4.26)	−**16.17 (6.42)**	−21.08 (11.36)	−**19.75 (4.32)**

*Positive values correspond to higher values for the Autonomous strategy. Bold values indicate statistically significant differences. Mean (SEM)*.

**Table 5:** Comparison of the Autonomous strategy to the Robot-Commands strategy for ratings reported by the participants and time taken for task completion in seconds.

	**Sort**	**Stack**	**Build**	**Balance**	**Overall**
Comfortable	0.75 (0.59)	0.00 (0.67)	0.50 (0.36)	0.08 (0.68)	0.33 (0.29)
Confused	0.33 (0.76)	1.08 (0.56)	0.75 (0.46)	0.50 (0.42)	**0.67 (0.28)**
Natural	**1.50 (0.57)**	1.08 (0.51)	**1.42 (0.53)**	0.83 (0.89)	**1.21 (0.31)**
Fluent	1.17 (0.61)	0.58 (0.68)	**1.33 (0.43)**	0.83 (0.61)	**0.98 (0.29)**
Efficient	1.83 (0.88)	0.42 (0.85)	0.75 (0.73)	0.42 (0.92)	**0.85 (0.42)**
Good Partner	1.08 (0.57)	−0.08 (0.71)	**0.92 (0.38)**	0.50 (0.80)	**0.60 (0.31)**
Time	−**49.67 (10.68)**	−**21.50 (4.09)**	−**35.08 (7.21)**	−**21.08 (2.99)**	−**31.83 (3.76)**

*Positive values correspond to higher values for the Autonomous strategy. Bold values indicate statistically significant differences. Mean (SEM)*.

**Table 6:** Comparison of the Human-Commands strategy to the Robot-Commands strategy for ratings reported by the participants and time taken for task completion in seconds.

	**Sort**	**Stack**	**Build**	**Balance**	**Overall**
Comfortable	0.75 (0.54)	0.33 (0.50)	0.50 (0.42)	0.08 (0.79)	0.42 (0.28)
Confused	0.08 (0.63)	0.42 (0.53)	0.33 (0.57)	0.75 (0.63)	0.40 (0.29)
Natural	**1.33 (0.40)**	**1.17 (0.37)**	**1.17 (0.37)**	0.83 (0.39)	**1.13 (0.19)**
Fluent	0.75 (0.55)	0.83 (0.39)	0.83 (0.51)	0.42 (0.58)	**0.71 (0.25)**
Efficient	1.42 (0.71)	0.50 (0.51)	0.67 (0.51)	0.58 (0.70)	**0.79 (0.30)**
Good Partner	0.33 (0.74)	0.00 (0.60)	0.42 (0.60)	0.17 (0.73)	0.23 (0.33)
Time	−15.83 (10.17)	−13.58 (2.68)	−**18.92 (2.42)**	0.00 (12.66)	−**12.08 (4.16)**

*Positive values correspond to higher values for the Human-Commands strategy. Bold values indicate statistically significant differences. Mean (SEM)*.

**Table 7:** Number of participants choosing each strategy as preferred for each task and overall.

	**Sort**	**Stack**	**Build**	**Balance**	**Overall**
Autonomous	8	5	6	3	6
Human-Commands	1	3	2	7	4
Robot-Commands	3	4	4	2	2

Two-tailed unpaired *t*-tests found that completion times for Sort, Build, and Overall were significantly shorter for the Autonomous strategy compared to the Human-Commands strategy, that completion times for all tasks and overall were significantly shorter for the Autonomous strategy compared to the Robot-Commands strategy, and that completion times for the Build task and overall were significantly shorter for the Human-Commands strategy compared to the Robot-Commands strategy.

We performed Wilcoxon Signed-Rank Tests on the differences in ratings between the interaction styles. No statistically significant differences were found between the Autonomous and Human-Commands strategy. The Autonomous strategy is rated as significantly more natural for the Sort and Build tasks, and as more fluent and as a better partner for the Build task when compared to the Robot-Commands strategy (*p* ≤ ‘0.05). The Human-Commands strategy is rated as significantly more natural for the Sort, Stack, and Build tasks when compared to the Robot-Commands strategy (*p* ≤ 0.05).

The results of the preferred interaction strategy overall and for each task indicates that this preference is very individual (see Table 7). Although each strategy was preferred by at least one participant for each task, there are clear trends in preference between the tasks. Eight participants preferred the Autonomous strategy for the Sort task, which reduced to five, six, and three for the Stack, Build, and Balance tasks. Seven participants preferred the Human-Commands strategy for the Balance task, citing their desire for control in completing this task. Overall, six participants preferred Autonomous, four preferred Human-Commands, and two preferred Robot-Commands. The participants who preferred Robot-Commands commented that they appreciated not needing to remember which block to place next.

#### 4.2.2. Discussion

The results of the Different Tasks study confirm three of our four hypotheses. Autonomous interactions were found to be more efficient for all tasks. While tasks involving joint action were not found to be perceived as more efficient in the human-led interactions, participants preferred Human-Commands for the Balance task as they wanted more control over joint actions. We found evidence for tasks with higher cognitive load being perceived as easier in robot-led interactions. Overall the robot-led interactions resulted in lower values for naturalness, fluency, efficiency, and whether the robot was a good partner.

The study identified two situations in which human-led or robot-led interactions might be preferred over autonomous interactions: participants prefer to have control over the interactions for tasks involving joint action and tasks that have higher cognitive load are perceived as easier in robot-led interactions.

### 4.3. Information experiment

For the Information experiment, we developed a strategy that acted as the Autonomous strategy previously, but also gave helpful commands and asked for timing commands from the human partner for joint actions. These improvements corresponded to situations from the previous experiments that were highlighted as requiring extra communication between the robot and the participant. Half of the participants performed the tasks with the Autonomous strategy first and the other half performed the tasks with the Information strategy first. The order of the four tasks is kept the same within each strategy in the order of increasing difficulty: Sort, Stack, Bridge, Balance. The participants rate the interactions on the same statements as the previous study, as well as a statement about how easy the task was:

- I was comfortable working with the robot
- I was confused working with the robot
- The interactions felt natural
- The interactions were fluent
- The interactions were efficient
- The robot was a good partner
- The task was easy

Our aim for this study was to develop a system that could be preferred over the Autonomous system. With the Information strategy developed to be useful for these tasks, we hypothesize that:

- The Autonomous and Information strategies have similar efficiency for these tasks.
- Tasks are perceived as easier with the Information strategy.
- Tasks are perceived as more confusing with the Autonomous strategy
- Participants prefer the Information strategy for all tasks and overall

#### 4.3.1. Results

We conducted a lab study with 10 participants (3 female) aged between 20 and 68 (*M* = 33.6, *SD* = 18.2). Two-tailed unpaired *t*-tests found that completion times for all tasks were significantly shorter for the Autonomous strategy when compared to the Information strategy. The responses were fairly similar across most statements for the different tasks and strategies (see Table 8, bold values correspond to statistically significant differences). We performed Wilcoxon Signed-Rank Tests on the differences in ratings between the interaction styles. The Autonomous strategy is rated as significantly more fluent overall compared to the Information strategy (*p* ≤ 0.05), but no other statistically significant differences were found between the Autonomous and Information strategies. However, for the Sort task, the Information strategy was rated as less fluent, less efficient, and the task was harder. For the Stack task, the Information strategy was rated as more natural, and for the Balance task, the Information strategy was rated as less confusing and the task was easier. Overall, the Information strategy was preferred by six of the ten participants (see Table 9). The Autonomous strategy was considered better for the Sort task by seven of the ten participants. Five participants preferred each strategy for the Stack task, and six participants preferred the Information strategy for both the Build and the Balance task.

**Table 8:** Comparison of the Autonomous strategy to the Information strategy for ratings reported by the participants and time taken for task completion in seconds.

	**Sort**	**Stack**	**Build**	**Balance**	**Overall**
Comfortable	0.20 (0.33)	−0.20 (0.20)	−0.10 (0.23)	−0.70 (0.45)	−0.20 (0.16)
Confused	−0.10 (0.55)	0.00 (0.00)	0.40 (0.31)	0.80 (0.53)	0.28 (0.21)
Natural	0.00 (0.98)	−1.00 (0.56)	−0.20 (0.57)	−0.50 (0.70)	−0.43 (0.35)
Fluent	1.00 (0.71)	0.60 (0.31)	0.40 (0.22)	0.20 (0.29)	**0.55 (0.21)**
Efficient	1.20 (0.63)	0.00 (0.42)	−0.30 (0.40)	−0.30 (0.50)	0.15 (0.26)
Good partner	0.00 (0.37)	0.30 (0.26)	0.60 (0.37)	0.30 (0.52)	0.30 (0.19)
Easy	0.60 (0.45)	0.00 (0.15)	−0.30 (0.26)	−0.80 (0.33)	−0.13 (0.17)
Time	−**17.50 (3.39)**	−**7.50 (1.30)**	−**9.90 (3.01)**	−**9.90 (2.15)**	−**11.20 (1.38)**

*Positive values correspond to higher values for the Autonomous strategy. Bold values indicate statistically significant differences. Mean (SEM)*.

**Table 9:** Number of participants choosing each strategy as preferred for each task and overall.

	**Sort**	**Stack**	**Build**	**Balance**	**Overall**
Autonomous	7	5	4	4	4
Information	3	5	6	6	6

All four of the participants who preferred the Autonomous strategy overall commented that they could see that the Information strategy could be useful if the tasks were more difficult or complicated. Three of these four participants performed the tasks with the Information strategy first, so had the extra experience of performing these tasks before interacting with the Autonomous strategy. Also, one of these participants chose the Information strategy as preferred for the Sort task, stating that they did so probably because it was the first task they performed with the robot, so the extra help was appreciated while they were working out what they were meant to be doing.

Five participants reported that they found the robot to be slow. Four participants commented that they liked it when the robot waited for them to say when to release the block for the Balance task. Five participants commented that for some tasks, it was useful to have the robot tell them what to do next, so that they did not have to work it out for themselves.

#### 4.3.2. Discussion

The results of the Information study show that the Autonomous mode is more efficient for all tasks, although the difference was smaller for the more complex tasks. Some participants found the tasks easier with the Information strategy and more confusing with the Autonomous strategy, and overall there is a trend that the Information strategy is preferred for more complex tasks. However, a limitation of this study was the difficulty of the tasks. While some participants found the tasks difficult enough to appreciate the assistance of the robot in the Information strategy, others found the tasks so simple that the extra information was unnecessary. We predict that the Information strategy would be preferred if this study was repeated with more complex tasks.

Despite this limitation, the study shows that the Information strategy is useful when completing tasks that participants consider difficult. In particular, the trend of the Information strategy being preferred more often for the more complex tasks of Build and Balance indicates the usefulness of the strategy in these situations. The qualitative feedback from the participants confirms that being told which action to take next is often appreciated and that controlling timing for joint action is also often desired. The key for future strategy improvement is for the robot to identify when its human partner considers a task difficult and therefore may appreciate extra information.

## 5. General discussion

In this paper, we investigated human-robot collaboration for on-table tasks. We presented a robot system for collaborating across the table from a human to build structures using colored blocks. Our first study confirmed that a robot acting in a Proactive or Autonomous manner is preferred over a robot acting Reactively (Baraglia et al., 2016). We found that the Proactive and Autonomous interaction styles were very similar for the bridge-building task, particularly as most actions were interleaved between the robot and human. We also found that the Human-Commands mode was preferred over the Human-Requested mode, as participants found it more natural to specify a particular action for the robot to take, rather than simply asking for help. The results from the second study also confirmed that the tasks are completed more efficiently when the participant and robot act autonomously. Tasks with fixed or partially fixed action order (Stack and Build) were rated as less confusing with robot-led interactions. Some participants indicated that they appreciated not needing to remember which block to place next for these tasks. Human-led interactions were preferred by the majority of participants for the task including joint actions (Balance), as they wanted to have more control for these actions. Our final study presented here confirmed that an interaction style that allows robot-led interactions for higher cognitive load actions and human-led interactions for joint actions can be preferred. The autonomous interactions were still preferred by approximately half of the participants, likely due to these tasks being easy for some people to complete without additional help from the robot.

We have confirmed our belief that different interaction styles are preferred for different tasks with respect to independent vs. joint action, and fixed vs. any order actions. It is therefore important to consider the type of task for designing robot interactions. For tasks involving joint action, including constructing furniture (Roncone et al., 2017) and complex structures (Munzer et al., 2017b), care should be taken to allow the robot to act autonomously for efficient task completion, but to allow the human to take control over interactions to allow for more natural collaborations. The Autonomous strategy was preferred for tasks involving independent actions that can be performed in any order (Sort). Collaborative clearing (Nikolaidis et al., 2017) and cleaning (Fiore et al., 2016) tasks can probably be efficiently executed with minimal communication between the agents. Our results indicate that tasks involving a set order of actions, including construction (Roncone et al., 2017), cooking (Milliez et al., 2016), and placement (Baraglia et al., 2016) tasks, would benefit from communication about the shared plan at the time when the human should take an action. However, as different people find different tasks easy or hard, a system that also adapts to the humans knowledge would be beneficial (Milliez et al., 2016). It is also important to consider how the findings from a “toy” task, such as the block building tasks used in this study, will be applicable in real world tasks (Mutlu et al., 2013). We believe that the effects found in our simple tasks here will be even more clear in more complex real world tasks involving actions with complicated dependencies and joint actions.

To answer the question: how do humans want to interact with collaborative robots? In the domain of table-top collaborative robots, interactions in which both humans and robots act autonomously result in more efficient interactions, but in joint action situations human-led interactions are preferred and in high cognitive load situations robot-led interactions are preferred. Specifically, joint actions benefit from timing information being communicated between the human and the robot, and in our studies, people preferred to provide this information. Additionally, actions that have a higher cognitive load benefit from the robot providing extra information for the human, by communicating its shared plan about what the human should be doing. A robot that can identify joint actions as well as actions that may require a higher cognitive load and act appropriately would result in both more efficient and more natural interactions.

## Ethics statement

The user study conducted in this work is exempt from approval at the University of Stuttgart, Germany. Prior to participating in this study, each participant signed a form giving explicit consent to video recordings of their interaction with the robot and to the use of these data for research purposes and research-related publication.

## Author contributions

All authors listed have made a substantial, direct and intellectual contribution to the work, and approved it for publication.

### Conflict of interest statement

The authors declare that the research was conducted in the absence of any commercial or financial relationships that could be construed as a potential conflict of interest.

## Footnotes

^1^http://wiki.ros.org/sound_play
